# Supplementary figures and images for: Endothelial Basement Membrane Laminins as an Environmental Cue in Monocyte Differentiation to Macrophages
Source: Front Immunol. 2020 Oct 30;11:584229. doi: 10.3389/fimmu.2020.584229 (PMC7662115; doi:10.3389/fimmu.2020.584229)

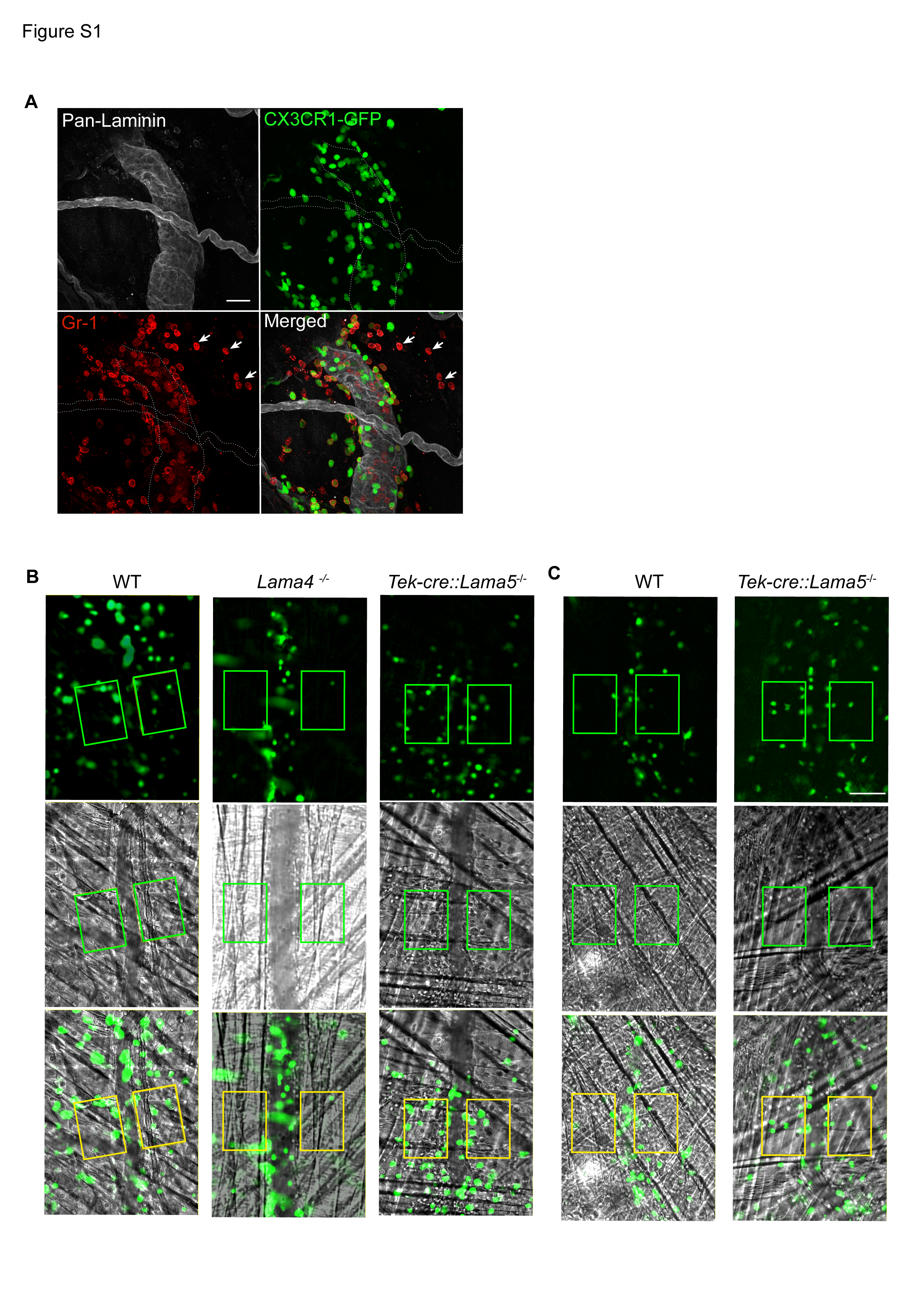

Supplement: Supplementary file 2 [file Image_1.tif]

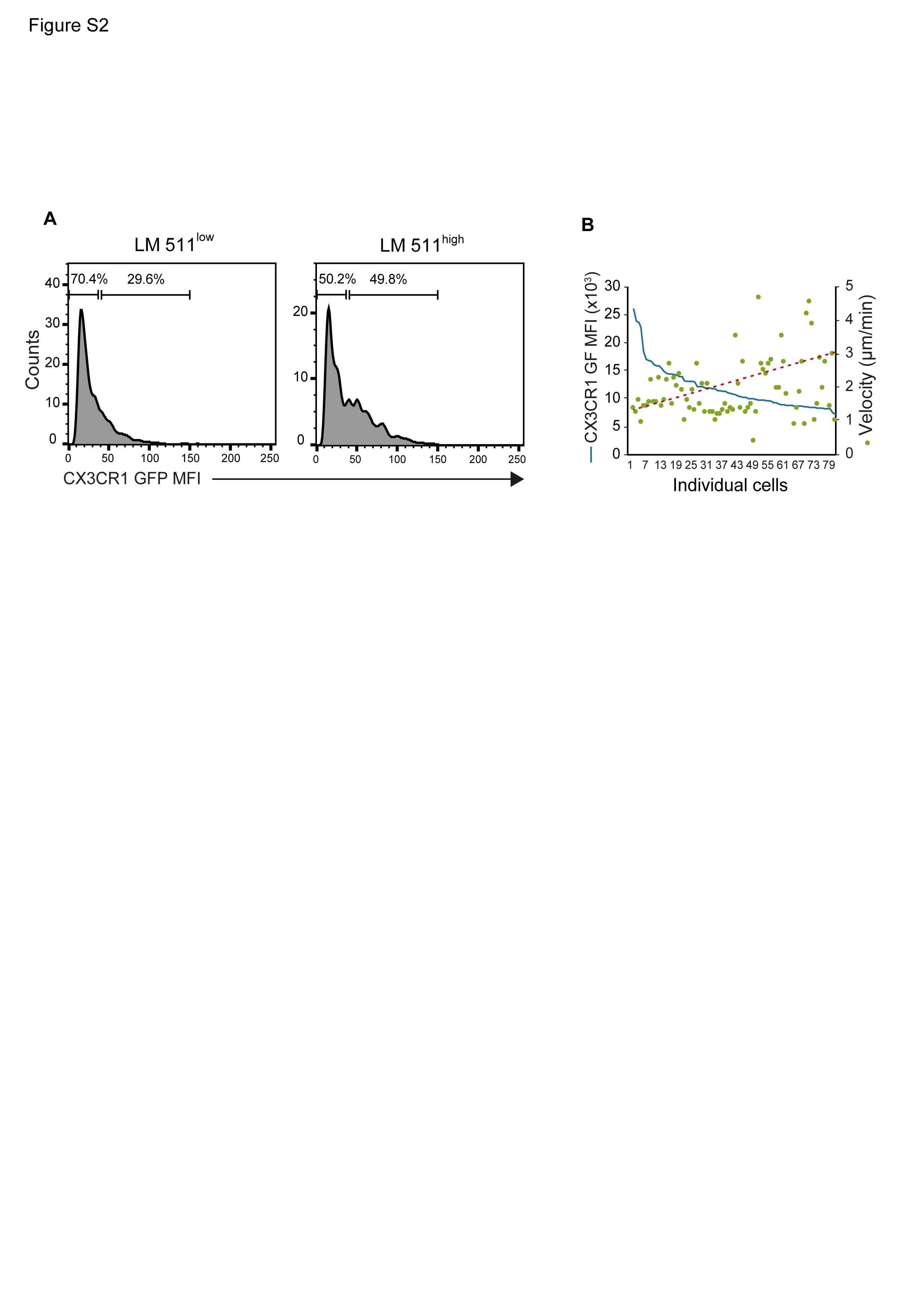

Supplement: Supplementary file 3 [file Image_2.tif]

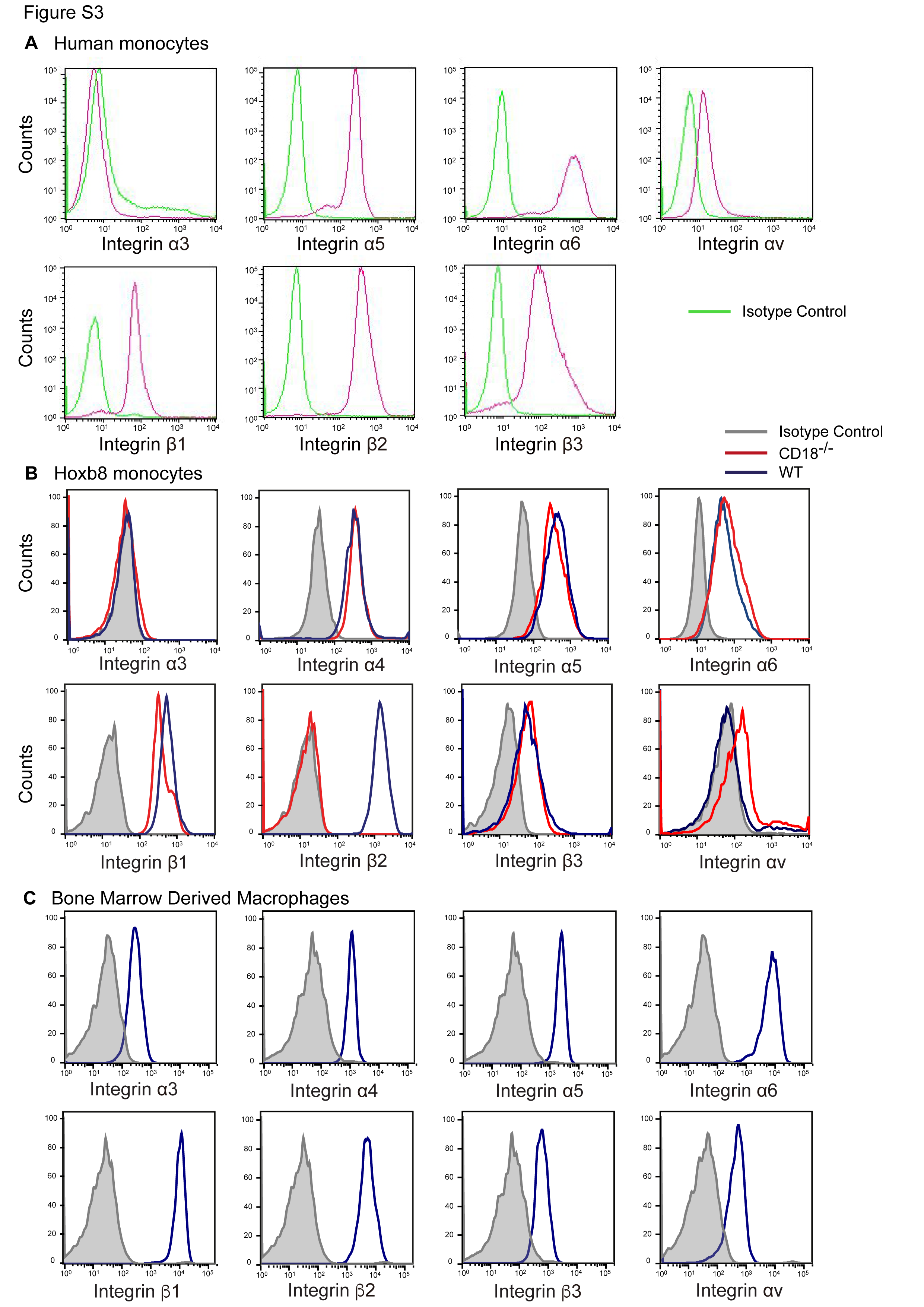

Supplement: Supplementary file 4 [file Image_3.tif]

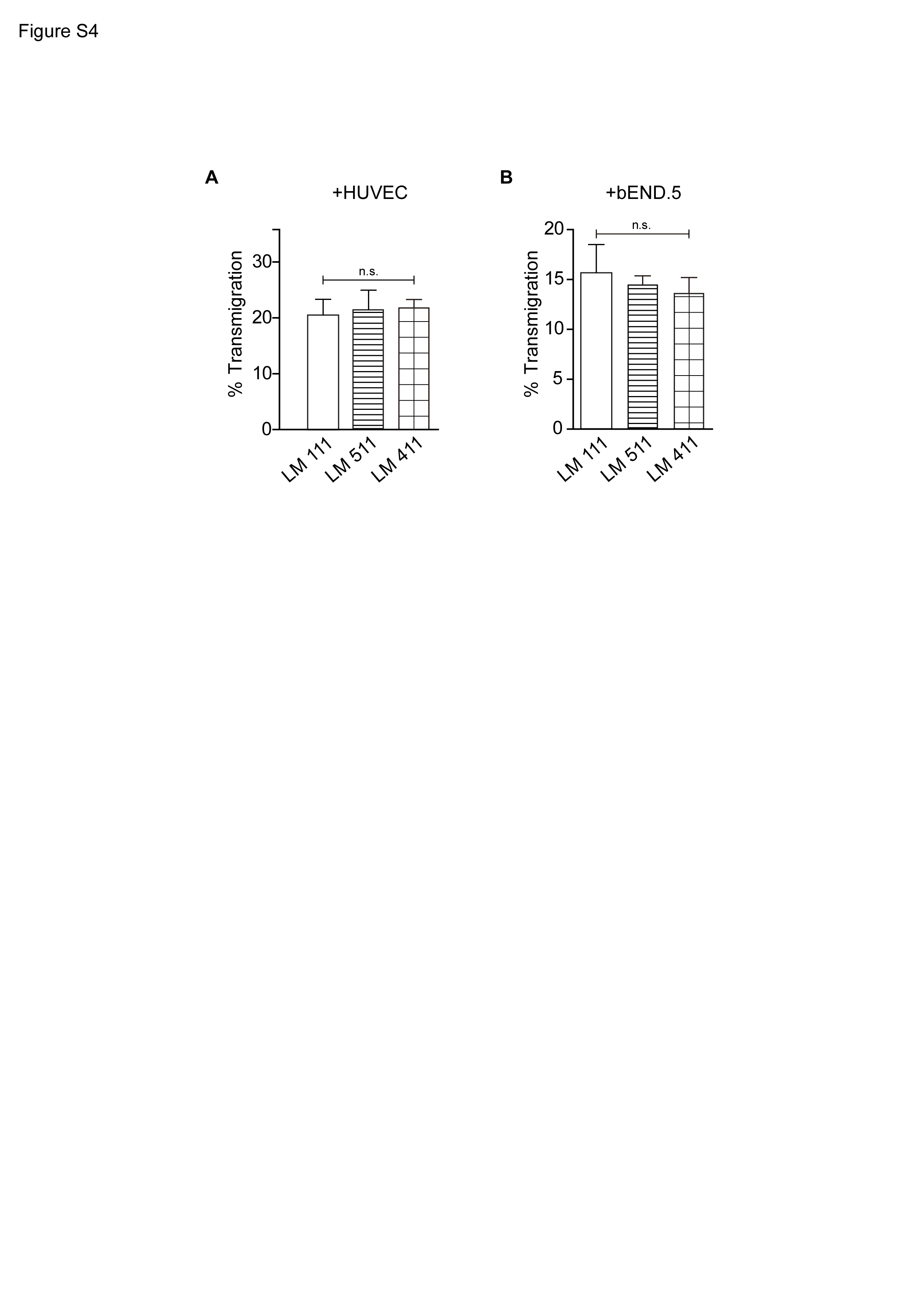

Supplement: Supplementary file 5 [file Image_4.tif]

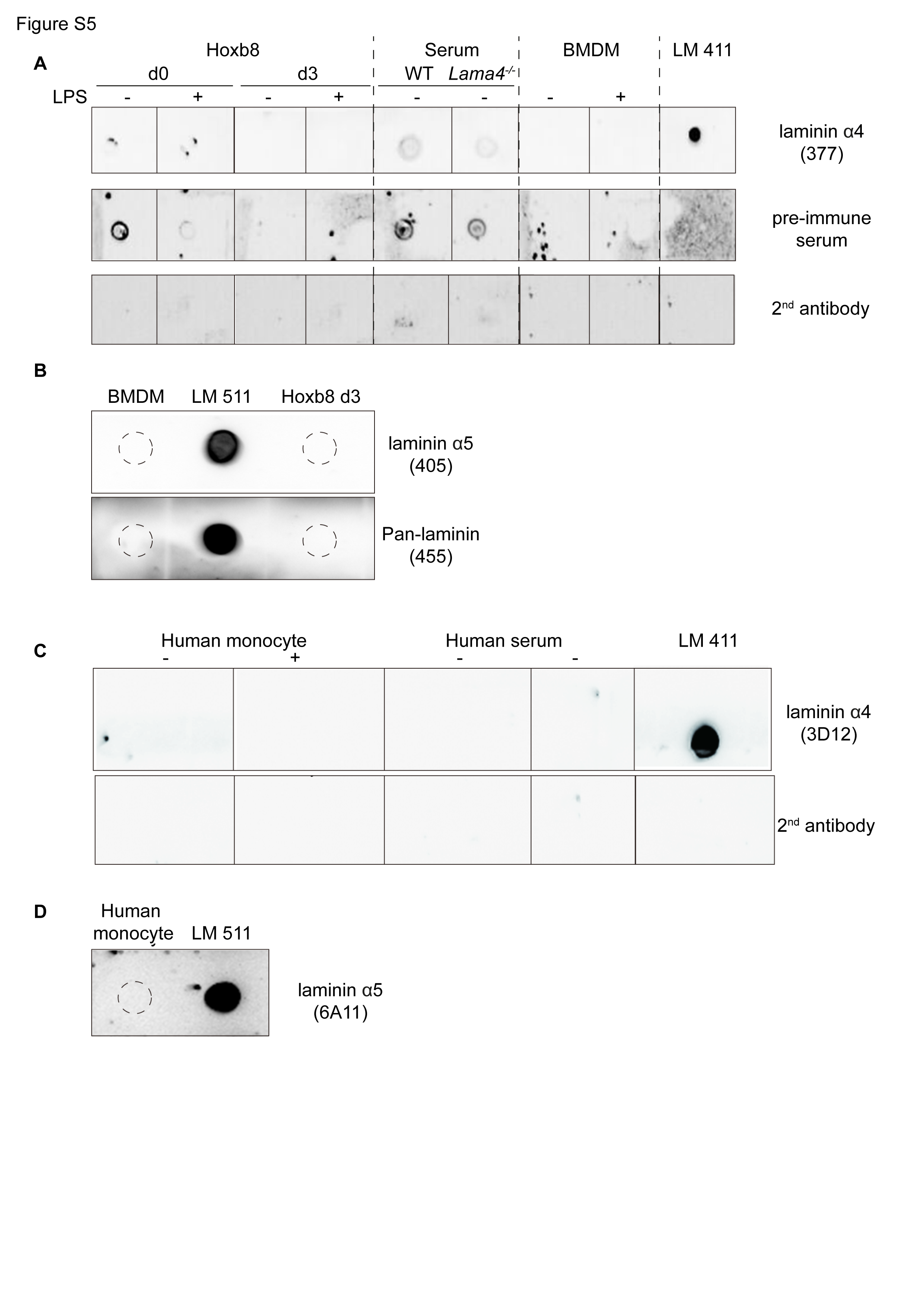

Supplement: Supplementary file 6 [file Image_5.tif]

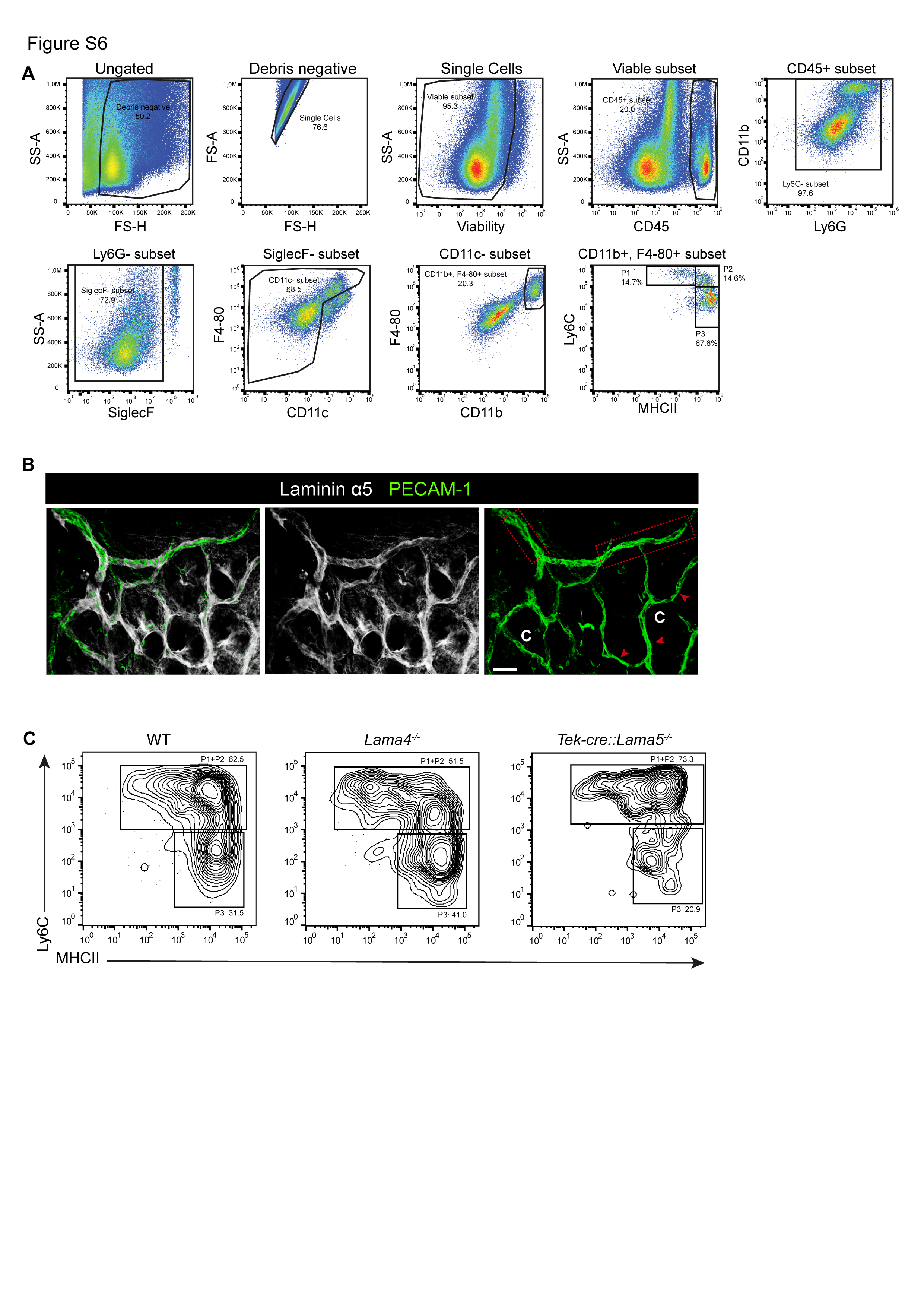

Supplement: Supplementary file 7 [file Image_6.tif]

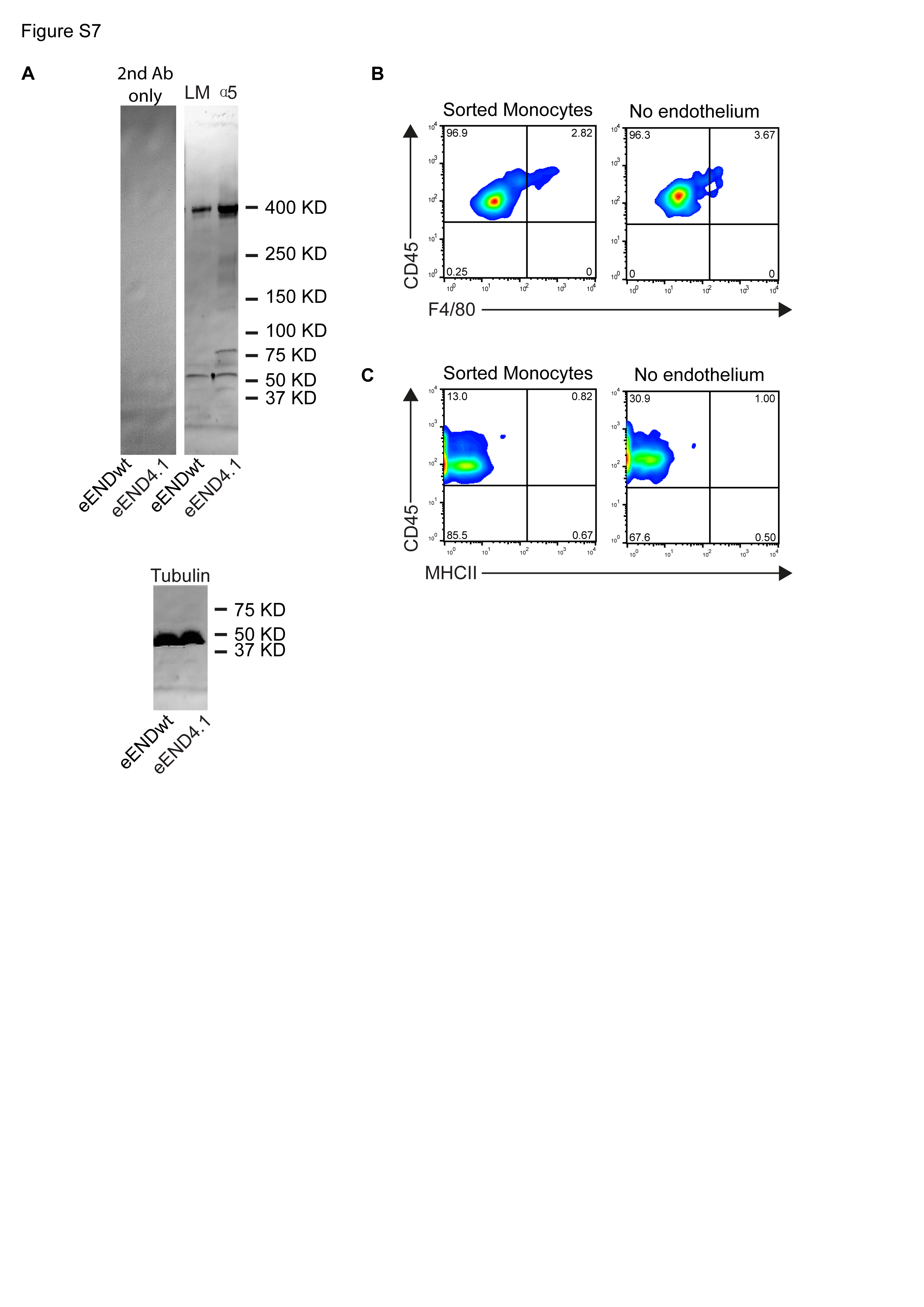

Supplement: Supplementary file 8 [file Image_7.tif]
